# A Computational Framework for High-Throughput Isotopic Natural Abundance Correction of Omics-Level Ultra-High Resolution FT-MS Datasets

**DOI:** 10.3390/metabo3040853

**Published:** 2013-09-25

**Authors:** William J. Carreer, Robert M. Flight, Hunter N. B. Moseley

**Affiliations:** Department of Molecular and Cellular Biochemistry, University of Kentucky, Lexington, KY 40536, USA; E-Mails: jim.carreer@uky.edu (W.J.C.); robert.flight@uky.edu (R.M.F.)

**Keywords:** stable isotope tracing, stable isotope-resolved metabolomics, Fourier transform mass spectrometry, multi-isotope natural abundance correction, analytical derivation, parallelization

## Abstract

New metabolomics applications of ultra-high resolution and accuracy mass spectrometry can provide thousands of detectable isotopologues, with the number of potentially detectable isotopologues increasing exponentially with the number of stable isotopes used in newer isotope tracing methods like stable isotope-resolved metabolomics (SIRM) experiments. This huge increase in usable data requires software capable of correcting the large number of isotopologue peaks resulting from SIRM experiments in a timely manner. We describe the design of a new algorithm and software system capable of handling these high volumes of data, while including quality control methods for maintaining data quality. We validate this new algorithm against a previous single isotope correction algorithm in a two-step cross-validation. Next, we demonstrate the algorithm and correct for the effects of natural abundance for both ^13^C and ^15^N isotopes on a set of raw isotopologue intensities of UDP-N-acetyl-D-glucosamine derived from a ^13^C/^15^N-tracing experiment. Finally, we demonstrate the algorithm on a full omics-level dataset.

## 1. Introduction

Stable isotope tracing has long been used to decipher pathways in cellular metabolism [1,2,3], with more recent applications permitting quantitative analyses of cellular metabolic processes [4,5]. The introduction of stable isotope-resolved metabolomics (SIRM) [5] and the use of Fourier transform mass spectrometers (FT-MS) have been instrumental in enabling the quantitative detection and tracing of metabolites [6,7,8]. These newer FT-MS instruments like the Kingdon-Makarov trap MS (OrbitrapTM) and Fourier transform-ion cyclotron resonance-MS (FT-ICR-MS) can achieve both ultra-high mass accuracies of better than 0.2 ppm (starting at 0.00008 Da @ 400 *m/z*) and ultra-high resolution of 400,000 or more (at 400 *m/z*, 10% valley). At these levels of accuracy and resolution, thousands of metabolites can be unambiguously detected and quantified as specific sets of isotopologue peaks. These sets of isotopologue peaks result from the ability to resolve the mass difference between isotopologues with the same nominal mass but differing isotope counts due to differing numbers of neutrons. Thus, an isotopically-resolved molecular formula can be determined for each isotopologue from its *m/z* ratio alone, when limited to isotopes in living systems. Combined with the ability to quantify the relative amount of each isotopologue, it becomes possible to trace the flow of metabolites and pathways [9], especially through the use of time-series experiments.

However, to be able to properly quantify the relative amount of each isotopologue in a SIRM experiment, the contribution of natural abundance (NA) must be factored out of each isotopologue peak. We previously reported the development of an algorithm specifically tailored for correcting FTMS SIRM isotopologue peaks [7]. While improving on prior numerical solutions designed for data from less accurate and less resolved mass spectrometers [10,11,12,13,14,15,16,17,18] and prior methods that did not address labeling [19,20,21], the implementation was a simple proof of concept script that took in a list of isotopologue peaks, the atom used for labeling, and the possible number of atoms that could be labeled in the formula.

The analysis of high-throughput metabolomics experiments requires the development of an integrated, high-performance system capable of performing the natural abundance correction on thousands of isotopologue peaks in a timely manner. Such development involves many considerations of computational and software architecture and best practices to have a working, easily extensible system. Below we describe the architecture of such a software system, verification of its correctness and its utility for performing natural abundance correction of large numbers of isotopologue peaks on reasonable timescales.

## 2. Methodology for Peak Correction

Correcting isotopologue peak intensities from ultra-high FT-MS experiments is accomplished using the previously derived equations from [7]. The full derivation is provided in the supplemental materials, however they are described here as they correspond to actual objects and methods used in the design of the software system.

As previously described, correction of the isotopologue intensities is performed on each isotopologue peak in turn, and iterated until convergence [7]. Equation (1) shows the full correction of each isotopologue peak of a triply labeled compound (C, N, and H). Note that this equation corrects the previously reported boundary limits of x, y, and z in [7], and expands the correction to systems where more than one of the elements is labeled.

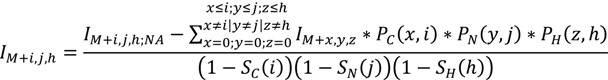
(1)

The P- and S-correction terms are generated separately for each element and possible combination of x and i. Equation (2) shows the calculation of the P-correction terms for ^13^C, while Equation (3) gives the calculation of the S-correction terms.

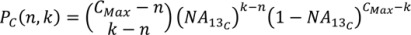
(2)

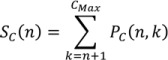
(3)

## 3. Software Design and Methods

### 3.1. Language and Library Choices

The Python scripting language [22] was chosen to implement the above equations in the form of a practical algorithm that is both efficient and parallelizable. Besides Python’s versatility in integrating diverse computational tools [23], the language offers several stable numerical modules that hastened the development of the software. The most important Python module used during development was numpy. This module provides a convenient and efficient class called an ndarray representing a simple *n*-dimensional array data structure, but with compiled execution speeds due to the module’s implementation in the C language [24]. These ndarrays are used in generating both P and S lookup tables (see below).

### 3.2. Data Flow

The software system takes a large collection of peak intensities that represent multiple molecular entities (data collection), and performs the correction on the set of peaks corresponding to the isotopologues of each molecular entity in turn (dataset). For a given data collection, a configuration must be defined that determines which columns contain relevant information such as the peak intensities, molecular formulas and isotopologue numbers, as well as the location of the input and output files. Initialization causes all the peak data to be read from the file and the generation of both P and S lookup tables as caches (see below for description of caching and data generated). Correction actually corrects each set of isotopologue peaks (see section 3.4 below for a description of the correction algorithm), using the previously cached values from the P and S lookup tables.

### 3.3. P and S Caching

To accommodate a general approach, Equations (2) and (3) are pre-computed before-hand and stored in 2D and 1D look up tables respectively, which we refer to as “P” and “S” tables. This is useful as the same value of P and S will be used multiple times to correct the peak intensities in a given dataset. However, it is important to note that the values calculated in Equations (2) and (3) are only dependent on the labeling element’s maximum count for a given molecule, and not the molecule itself. Therefore, the same pre-computed values can be used for any molecules with the same number of atoms for the natural abundance correction element. Moreover, many experimental datasets include replicate and time series entries for the same molecule, which augments the utility of caching these tables.

### 3.4. Correction Algorithm, Constructors, and Modularity

**Figure 1:**
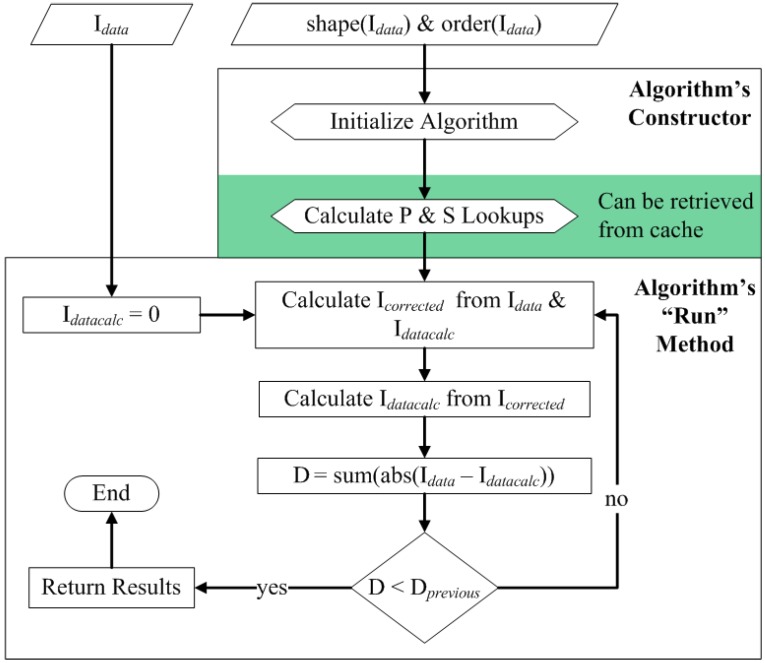
Procedural diagram of the isotopic natural abundance correction algorithm. Starting with the shape and order of the set of observed isotopologues, the algorithm is initialized, followed by the calculation of the P and S tables or their recovery from a cache. Next, the corrected isotopologue intensities (I*_corrected_*) are calculated from the observed isotopologue intensities (I*_data_*). Then the isotopical natural abundance contaminated intensities (I*_datacalc_*) are calculated from the corrected intensities. The calculated and observed intensities are compared. If an improvement is made, the calculation cycle is repeated.

The implementation of Equation (1) encompasses a general strategy that is applicable for 1, 2 or 3 labeling sources (see the derivation in the supplement for equations for 1 and 2 labeling sources) as well as to the correction for natural abundance for any list of isotopes used in a multi-isotope labeling scheme. Figure 1 shows the general flow of the algorithm. The algorithm iteratively refines the natural abundance correction via a series of additions and subtractions of isotopic natural abundance from the dataset for labeling isotopes. This iterative approach decreases the propagated error by half [7]. In order to accommodate any possible list of labeling isotopes, the algorithm was developed as a Python class called NACorrector, with similarities to both “abstract factory” and “template method” design patterns [25], but where object creation implements a specific algorithm. Given the dynamic nature of Python objects, object instantiation can have similarities to concrete class implementation in more rigid languages. An instance of this NACorrector class must be initialized for a particular dataset before being used. This initialization is handled in the constructor method.

The shape and order of I*_data_* are tuples of length *n*, where *n* is the number of labeling sources used in the experiment (*i.e*., the data’s dimensionality). The data’s shape defines the maximum isotope count for each labeling source (*i.e.*, C_Max_ for ^13^C), while its order defines which isotope label corresponds to which dimension. For example, a shape of (23,54) and an order of (“15N”,”13C”) corresponds to the dataset of a molecule with 23 nitrogen atoms and 54 carbon atoms respectively and indicates that ^15^N and ^13^C were used as labels. The P and S lookup tables are calculated based on the dataset’s order and shape, however if these tables are supplied to the algorithm’s constructor via a cache (see Section 3.3), these calculations will be skipped. Another advantage of using an algorithm-object design is that each instance of the algorithm can be optimized at runtime, depending on the dataset’s dimensionality. Specifically, the overhead necessary for converting a multidimensional index to a flat index is not required when the algorithm is operating on a dataset where only a single isotopic label was used. The implementation takes this into account in the algorithm’s constructor method and uses a much faster indexing function when operating on a one-dimensional dataset.

**Figure 2:**
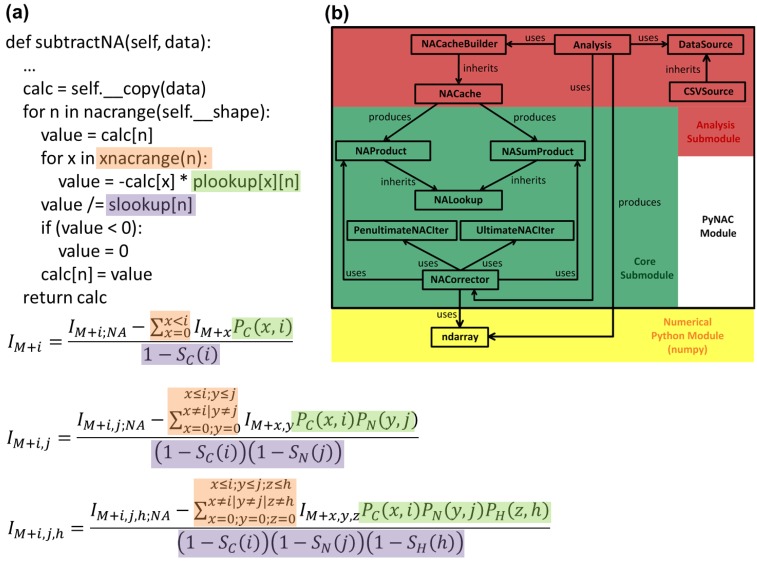
Algorithm generalization and class relations in the modularization of the code. (**a**) The orange xnacrange object is generalized to handle the different summations in each formula. The light green plookup object generalizes the P table representing different sets of binomial terms specific to each formula. The purple slookup object generalizes the S table representing different sets of summative binomial terms specific to each formula. (**b**) The PyNAC module has several classes separated into the green Core submodule or the red Analysis submodule. The blue multiprocessing module is provided by the standard Python language library. The yellow numpy module is the only additional python library that is necessary. The NACorrector class implements the main correction algorithm using the ndarray class from the nympy module. The PenultimateNACIter, NAProduct, and NASumProduct classes implement the orange xnacrange, light green plookup, and purple slookup objects.

To increase maintainability and to reduce the complexity of the code base that represents this algorithm, the proper functions for iteration are determined during NACorrector’s object initialization (in the constructor method). If the algorithm is initialized to operate on datasets with only a single labeling source, the standard python function “range” is used. However, if the algorithm is initialized to operate on datasets that have multiple labeling sources, the penultimate and ultimate iteration functions replace the use of the range function where appropriate. The P and S lookups used in the algorithm are also tailored to the dimensionality of the datasets undergoing natural abundance correction and operate in tandem with the penultimate and ultimate iteration functions. Figure 2a shows how this initialization behavior can be used to keep the generalized form of the algorithm the same for any labeling configuration. Keep in mind that the index *x* and *n* can represent either multidimensional tuples or a single integer, depending on how the algorithm was initialized.

Here, if the algorithm is to operate on a multidimensional dataset, the function pointers for “nacrange” and “xnacrange” are replaced with the ultimate and penultimate iteration functions respectively. This occurs only once, during the initialization phase of the algorithm and doing so reduces the logical complexity of the calculations immensely. If the algorithm is initialized for data with only one labeling source (*i.e*., one dimensional) then these function pointers are replaced with python’s built in range function. The P and S lookups have also been initialized to reflect the dimensionality of the dataset, and perform the correct mathematical calculations internally based on the indices passed to them during access. Because many methods are reused in multiple places, the implementation is organized into modules and classes to encapsulate functionality. Figure 2b shows the basic layout of these modules and the various relationships between the classes found within.

The PyNAC (black square) module encompasses all classes and functions related to our implementation, however only the Core submodule (green) implements the actual algorithm. NACorrector is the actual algorithm class, and it is supported by NAProduct and NASumProduct, which are classes that represent the P and S lookup tables respectively. PenultimateNACIter and UltimateNACIter are special iteration classes that return tuple indices describing a location in a multidimensional array. Each takes a stopping criterion: a tuple of length *n* where *n* is the dimensionality of the array being traversed. The members of this tuple represent the maximum value each dimension of the returned indices can take during iteration. As the name suggests, the penultimate iterator returns all the indices within the penultimate set of this iterator’s stopping criterion, while the ultimate iterator returns all indices including the stopping criterion itself. These classes are replaced with Python’s built in range function to increase iteration efficiency when operating on a 1-dimensional dataset.

### 3.5. Quality Control

Included in the correction analysis are several data quality control measures. First, the data read from an input file is checked to ensure that each peak conforms predictably to the specifications of the configuration. These checks include insuring that the isotope count for a given peak does not exceed the maximum number of atoms for that element specified by the peak’s molecular formula. Second if two peaks with the exact same isotopic composition are found to belong to the same data set, the second peak is flagged as being a duplicate. If a particular peak fails one of these checks, the specific error message related to it is appended at the end of its row in the output file. Generally these errors occur when the correction analysis has been misconfigured, however they could also occur in data files that have been corrupted.

In addition to these basic checks, the correction analysis also allows for the configuration of a predicted peak inclusion threshold. This threshold can be defined as a percent of the minimum, maximum, or average peak intensity for the entire data collection or for each data set individually. If a peak is predicted at or above this threshold value, but not observed in the original data, the peak is added to the output file with special notation to alert researchers that the peak was predicted above the specified threshold but not observed in the data file they supplied. The inclusion of these predicted peaks is an important secondary check for researchers. Peak identification must be carried out before natural abundance correction. However, alerting the researcher that there are significant peaks predicted, but missing from the data collection, can insure better data quality. If, for example, many peaks are predicted but missing across many data sets in the data collection, a researcher may re-evaluate her methods of peak identification, and subsequently go back to the raw FTMS data to either identify the missing peaks manually or relax the restrictions for the software identifying the peaks.

### 3.6. Implementations of Binomial Terms

The calculation of the P correction terms is implemented as an interleaving for-loop constructed in such a way as to emulate a full expansion of the binomial term and the exponents, see Equation (2), while mitigating some of the effects of multiplying very large and small double precision values together. To verify that the interleaving (org) does in fact mitigate these types of errors, alternative methods for calculating P correction terms were tested using: (i) “factorials” from Pythons math module (choose); (ii) the “comb” function from SciPy, which is an “exact” multiplicative calculation (comb); (iii) the “log-gamma” function from SciPy (comb2); and (iv) a log10 version of the algorithm (logReal). P correction terms for the full range of n and k using an iMax of 500 and the natural abundance of deuterium (0.00015) were generated using each of the methods. Relative differences between all of the methods were calculated. Supplemental material contains all of these implementations.

### 3.7. Cell Culture and FT-ICR-MS

The singly labeled ^13^C data is from glycerophospholipids separated from crude cell extracts derived from MCF7-LCC2 cells in tissue culture after 24 h of labeling with uniformly labeled ^13^C-glucose. The doubly labeled ^13^C/^15^N data is from polar metabolites separated from crude cell extracts derived from MCF7-LCC2 cells in tissue culture after 24 h of labeling with uniformly labeled ^13^C/^15^N glutamine. Samples were directly infused in positive (glycerophospholipid) and negative (metabolites) ion modes on a hybrid linear ion trap 7T FT-ICR mass spectrometer (Finnigan LTQ FT, Thermo Electron, Bremen, Germany) equipped with a TriVersa NanoMate ion source (Advion BioSciences, Ithaca, NY, USA), with peaks identified as previously described [6].

## 4. Results and Discussion

### 4.1. Validation of the Algorithm

We used a progressive approach to cross-validate, in the analytical sense, all parts of both the single- and multi-isotope implementations of the algorithm. First, we performed a cross-validation between the single-isotope Python implementation and the original single-isotope Perl implementation from Moseley, 2010 [7]. Both real and simulated datasets were used in this cross-validation. We took the absolute difference between the values from the results of each implementation and checked that each value was below an acceptable threshold. Table 1 shows a comparison between the old single-isotope Perl implementation and the new single-isotope Python implementation. The test set describes a sample of the metabolite UDP-GlcNAc (C_17_H_27_N_3_O_17_P_2_). In all cases, the difference between the original implementation and the new implementation were either zero or insignificant (*i.e.*, <10^−9^). Furthermore, this approach cross-validates all parts of the iterative single-isotope Python implementation at once, including both the subtractNA and addNA functions.

**Table 1:** Comparison of the old Perl and new Python single-isotope algorithm implementations using isotopologues of UDP-GlcNAc.

^13^C Count ^a^	Intensity ^b^	Python (New) ^c^	Perl (Old) ^d^	Difference
5	187.9	214.81	214.81	2.27 × 10^−^^10^
6	60.5	39.81	39.81	1.79 × 10^−^^11^
7	109.8	116.15	116.15	1.78 × 10^−^^10^
8	418.4	449.36	449.36	3.58 × 10^−^^10^
9	23.1	0	0	0
10	165	176.39	176.39	3.68 × 10^−^^10^
11	1438	1,523.77	1,523.77	2.63× 10^−^^9^
12	1,215.9	1,183.78	1,183.78	3.59 × 10^−^^9^
13	4,235.8	4,360.57	4,360.57	3.63 × 10^−^^9^
14	1,562.5	1,420.73	1,420.73	2.17 × 10^−^^9^
15	1,253.9	1,231.68	1,231.68	4.81 × 10^−^^9^
16	175.8	149.9	149.9	4.44 × 10^−^^10^

^a^ Zero valued isotopologue intensities have been omitted from the table for the sake of brevity; ^b^ Observed uncorrected isotopologue intensities; ^c^ Corrected intensities using the Python implementation; ^d^ Corrected intensities using the older Perl implementation.

Now the multi-isotope Python implementation is cross-validated against the single-isotope Python implementation via the creation and use of a simulated multi-isotope isotopologue intensity dataset. Table 2 shows simulated ^13^C and ^15^N single-isotope datasets, each with normalized isotopologue intensities that sum to 1 and each representing a molecule with 9 carbon atoms and 6 nitrogen atoms, respectively.

**Figure 3:**
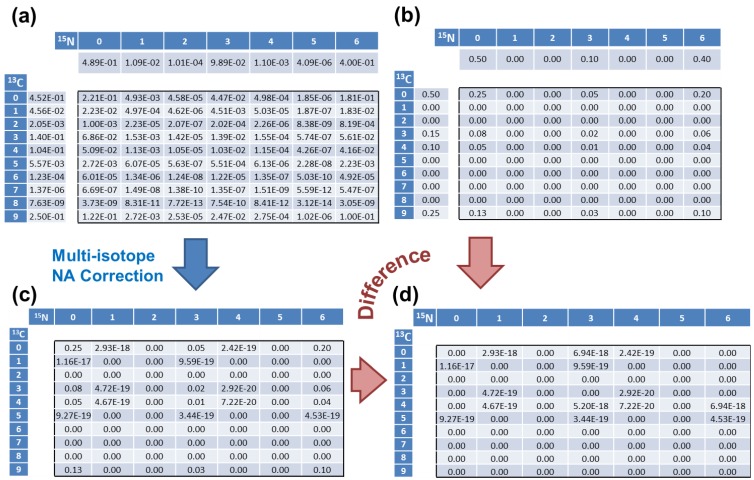
Validation of the multi-isotope natural abundance correction algorithm. (**a**) The matrix outlined by the black lines represents isotopologue intensities with ^13^C and ^15^N isotopes from both a labeling source and natural abundance. It is calculated from the vector outerproduct of two single isotope labeled vectors of isotopologue intensities. These single-labeledvectors represent the addition of ^13^C/^15^N natural abundance to the corresponding single-labeled vectors in (**b**). Each vector and matrix of intensities is normalized to a sum of 1. (**b**) The matrix outlined by the black lines represents isotopologue intensities with ^13^C and ^15^N isotopes from only a labeling source. It is calculated from the vector outer product of two single isotope labeled vectors of isotopologue intensities. (**c**) This matrix is the result of just one iteration of the multi-isotope natural abundance correction algorithm implemented in the Python programming language. (**d**) This matrix is the absolute difference between the matrices in (**b**) and (**c**). No element is larger than 10^−17^.

Using the validated addNA function from the single-isotope Python implementation, we added the effects of natural abundance to both a ^13^C simulated dataset and a ^15^N simulated dataset, with the results also shown in Table 2. Next, we calculated the vector outer products of both the simulated datasets and the simulated natural abundance tainted datasets to produce matrices representing a multi-isotope isotopologue intensity dataset for a molecule with both 9 carbon and 6 nitrogen atoms, as shown in Figure 3a,b respectively. Then, we applied the multi-isotope Python implementation to the matrix in Figure 3a to produce the natural abundance corrected matrix in Figure 3c. Next, we took the absolute difference between the matrices in Figure 3b,c, which is shown in Figure 3d. All of the specific differences in the Figure 3d matrix elements were either zero or below 10^−16^. Also, this approach cross-validates all parts of the iterative multi-isotope Python iteration at once, including both the specific subtractNA and addNA functions. Furthermore, these results demonstrate the numerical stability of the multi-isotope implementation, even though the algorithm is dealing with NxM data points and not just N data points.

**Table 2:** Simulated 13C and 15N single-isotope isotopologue intensity datasets.

**^13^C Count**	**0**	**1**	**2**	**3**	**4**	**5**	**6**	**7**	**8**	**9**
**Simulated**	0.5	0	0	0.15	0.1	0	0	0	0	0.25
**addNA**	0.4523	0.0456	0.0020	0.1403	0.1040	0.0056	1.2 × 10^−^^4^	1.4 × 10^−^^6^	7.6 × 10^−^^9^	0.25
										
**^15^N Count**	**0**	**1**	**2**	**3**	**4**	**5**	**6**	**-**	**-**	**-**
**Simulated**	0.5	0	0	0.1	0	0	0.4	-	-	-
**addNA**	0.4890	0.0109	0.0001	0.0989	0.0011	4 × 10^−^^6^	0.4	-	-	-

Note: The addNA rows are the results produced by the addNA function from the single-isotope Python implementation. Each row of values is normalized to a sum of 1.

### 4.2. Numerical Analysis of Interleaving Method

The P correction terms generated using the interleaving method were compared to alternative implementations of Equation (2) (see Methods) using various methods, including the original interleaving (org), factorials (choose), SciPy combinatorials (comb, comb2), and a log-version of the interleaving algorithm (logReal). The full set of pairwise differences is shown in Table 3. The maximum difference between any two methods was 5.7 × 10^−14^, well below any level that one would call significant in double precision math. The “choose” and “comb” methods gave identical values throughout, likely due to Pythons use of “long” integers with arbitrary precision. The “comb2” method gave small differences, as the log-gamma method is less accurate than using factorials or multiplicative formulations of the binomial. For a discussion of the stability of the interleaving method see the supplemental materials.

**Table 3:** Maximum differences between each of the methods used to calculate the *P* correction values.

	org	comb	comb2	choose	logReal
**org**	0	−2.36 × 10^−^^16^	−5.67 × 10^−^^14^	−2.36 × 10^−^^16^	−2.36 × 10^−^^15^
**comb**	-	-	−5.66 × 10^−^^14^	0	−2.25 × 10^−^^15^
**comb2**	-	-	-	5.66 × 10^−^^14^	5.48 × 10^−^^14^
**choose**	-	-	-	-	−2.25 × 10^−^^15^

### 4.3. Application to Observed Isotopologues of UDP-GlcNAc

**Figure 4:**
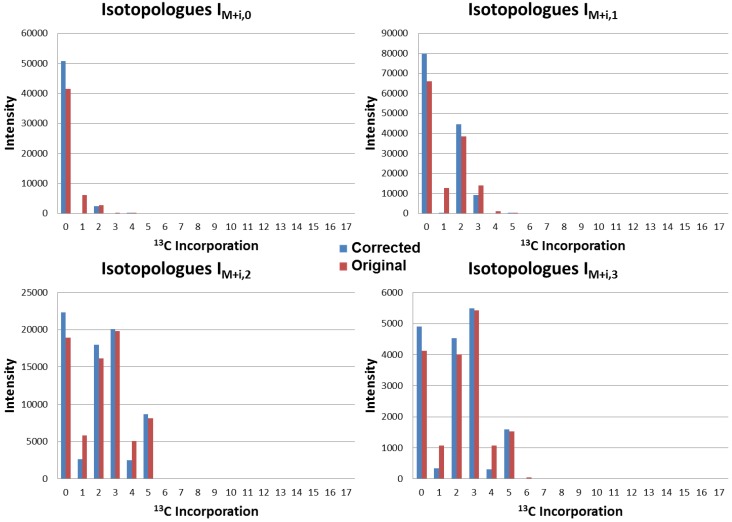
Corrected and observed ^13^C/^15^N isotopologues of UDP-GlcNAc. Each graph represents a set of ^13^C-labeled isotopologues with a specific number of ^15^N nuclei incorporated. I_M+i,0_, I_M+i,1_, I_M+i,2_, and I_M+i,3_ represent 0,1,2, and 3 ^15^N nuclei. Observed intensities are in red and the isotopic natural abundance corrected intensities are in blue. The calculation of the corrected intensities required 12 iterations of the algorithm.

Figure 4 shows the application of the multi-isotope Python implementation applied to a real dataset of ^13^C/^15^N isotopologue intensities for uridine diphosphate-N-acetyl-D-glucosamine or UDP-GlcNAc (C17H27N3O17P2). There are significant changes in quite a few of the isotopologues, but especially I_M+1,0_, I_M+1,1_, I_M+1,2_, I_M+4,2_, I_M+1,3_, and I_M+4,3_, where I_M+i,j_ refers to the incorporation of i ^13^C nuclei and j ^15^N nuclei. In fact, the effects of isotopic natural abundance are more dramatic than what is seen for single labeling experiments. This is to be expected since the effects of isotopic natural abundance for two elements is naturally greater than for either element.

### 4.4. Running Time

To test the effect of caching the P and S table calculations on the running time of the software, we ran it both in a default mode where caching is enabled, and alternatively forcing the recalculation of the P and S tables for each data set of peaks. With caching enabled, the run time averaged 530 s (9 min). Without caching enabled, the run time averaged 890 s (15 min). Both runs were performed on an Intel^®^ Xeon X5650 processor running at 2.67 GHz. Also, these timings used a data file of 9,066 different metabolites, with an average of 5 isotopologue peaks per metabolite.

## 5. Conclusions

Correction for the effects of natural abundance for multiple isotopes simultaneously is both computationally feasible and numerically stable when the raw isotopologues are isotopically resolved and identified. In addition, our algorithm is numerically stable both with respect to increasing isotope incorporation and to increasing dimensionality of the correction due to multiple isotopes. In addition, these corrections of isotopologue intensities are required before further quantitative analyses can be applied to SIRM experimental datasets, especially for determining metabolic flux. In general, SIRM experiments can generate massive volumes of data in relatively short periods of time. A single experimental dataset may contain well over 100,000 isotopologue intensities and can be collected in as little as five minutes with current FT-ICR mass spectrometers, like the one described in the Methods section. This makes natural abundance isotopic correction a high throughput computational problem. Fortunately, our current algorithm can correct for natural abundance on this time scale (*i.e.*, ~5 min data collection *vs* ~9 min analysis), with tools to detect and help correct issues in data quality. However, since each isotopologue set's correction calculations are independent from all others within the entire dataset, many sets could be corrected simultaneously. The design of the software inherently allows for a multi-processor implementation that scales to any number processor cores available, making the algorithm extremely robust with regard to future multi-core improvements in computing hardware. In addition, utilization of multiple processor cores will be required when applying this algorithm to error analysis of large datasets in a timely manner [26].

## Software Availability

The software system is available as a tarball in the supplemental materials, and includes an example of running the software on a file to correct multiple compounds in a single file. The software may also be downloaded from [27].

## Supplementary Files

Supplementary File 1 Supplementary Materials (ZIP, 1100 KB)
