# Supplementary material for: A Computational Framework for High-Throughput Isotopic Natural Abundance Correction of Omics-Level Ultra-High Resolution FT-MS Datasets
Source: Metabolites. 2013 Sep 25;3(4):853–66. doi: 10.3390/metabo3040853 (PMC3882318; doi:10.3390/metabo3040853)
Supplement: Supplementary File 1 — Supplementary Materials (ZIP, 1100 KB) [file metabolites-03-00853-s001.zip › metabolites-03-00853-supplementary/Supplemental Equations.pdf]

Supplemental Materials

Equation (1)

$$I_{M+i,j,h} = \frac{I_{M+i,j,h;NA} - \sum_{x=0;y=0;z=0}^{x\neq i|y\neq j|z\neq h} \overset{x\leq i;y\leq j;z\leq h}{} I_{M+x,y,z} * P_C(x,i) * P_N(y,j) * P_H(z,h)}{(1 - S_C(i))(1 - S_N(j))(1 - S_H(h))}$$

Equation (2)

$$P_C(n,k) = \binom{C_{Max} - n}{k - n} (NA_{13_C})^{k-n} (1 - NA_{13_C})^{C_{Max}-k}$$

Equation (3)

$$S_C(n) = \sum_{k=n+1}^{C_{Max}} P_C(n,k)$$
