# Supplementary material for: A Computational Framework for High-Throughput Isotopic Natural Abundance Correction of Omics-Level Ultra-High Resolution FT-MS Datasets
Source: Metabolites. 2013 Sep 25;3(4):853–66. doi: 10.3390/metabo3040853 (PMC3882318; doi:10.3390/metabo3040853)
Supplement: Supplementary File 1 — Supplementary Materials (ZIP, 1100 KB) [file metabolites-03-00853-s001.zip › metabolites-03-00853-supplementary/Supplemental Materials.pdf]

# Supplemental Materials

## 1. Derivation of Isotopologue Correction

### *1.1. Single Isotope Label*

Equation (1) is a generalized representation of the relative distribution of carbon (C) isotopologues from natural abundance only. In this equation $I_{M+0}$ represents the theoretically untainted $^{12}C$ monoisotopic peak. The term $I_{M+i;NA}$ represents the expected intensity of the *i*th isotopologue peak containing *i* $^{13}C$ atoms.

$$I_{M+i;NA} = I_{M+0}\left[\sum_{j,k}^{\substack{j,k\geq 0\\ j+2k=i}} \binom{C_{Max}}{j, k, C_{Max}-j-k} \left(NA_{13_C}\right)^j \left(NA_{14_C}\right)^k \left(NA_{12_C}\right)^{C_{Max}-j-k}\right] \quad (1)$$

The terms $NA_{x_C}$ represent the fractional natural abundance of the *x* isotope of carbon. The number of carbons in the molecule is represented by $C_{Max}$. This equation uses the multinomial theorem with 3 variables to express the number of isotopomers of identical mass for a molecule with $C_{Max}$ carbons given the 3 isotopes of carbon: $^{12}C$, $^{13}C$, and $^{14}C$. Because $^{14}C$ is extremely rare, FT-MS peaks containing this isotope are not observed for charged molecules from living systems, making its contribution to this calculation negligible. In addition, the very high resolution of the FT-IRC-MS histograms allows for complete deconvolution and identification of isotopologue peaks representing molecules exclusively comprised of the expected isotopes found in biological systems (primarily isotopes of CHONPS elements) and $^{13}C$. Equation (2) is a simplified form of Equation (1) that takes these facts into account. Removing $^{14}C$ simplifies the equation to a single isotopic fractional term and a binomial coefficient. In this case the binomial coefficient represents the number of possible isotopomers of identical mass for a molecule with $C_{Max}$ carbons given only 2 isotopes of carbon: $^{12}C$ and $^{13}C$. *i* is the number of $^{13}C$ in that isotopologue peak.

$$I_{M+i;NA} = I_{M+0}\binom{C_{Max}}{i}\left(NA_{13_C}\right)^i\left(1-NA_{13_C}\right)^{C_{Max}-i} \quad (2)$$

Equation (2) outlines the relationship between each peak, $I_{M+i;\,NA}$ and the theoretically untainted $^{12}C$ monoisotopic peak, $I_{M+0}$, which will have a fractional intensity of 1 when dividing by the sum of the isotopologue intensities. However, the calculation of contributions due to natural abundance becomes more complex when the introduction of $^{13}C$ from a labeling source is taken into consideration [1,2]. The effect of $^{13}C$ natural abundance is now related to the amount $^{13}C$ already present due to labeling. The solution to this dilemma is to use a series of binomial terms to correct for $^{13}C$ natural abundance for each $^{12}C/^{13}C$ isotopologue resolved in the mass spectrometer histogram based on every other $^{12}C/^{13}C$ isotopologue present. Equation (3) describes the combinatorial part of these terms as a function of *n* and *k*, where *k* represents the total number of $^{13}C$ carbons present, *n* represents the number of $^{13}C$ carbons present due to the labeling source, and *k*-*n* represents the number of $^{13}C$ carbons present due to $^{13}C$ natural abundance [3].

$$P_C(n,k) = \binom{C_{Max} - n}{k - n} \left(NA_{13_C}\right)^{k-n} \left(1 - NA_{13_C}\right)^{C_{Max}-k} \tag{3}$$

Here the binomial coefficient is used to enumerate the number of ways that $k - n$ $^{13}C$ can be incorporated due to natural abundance into a molecule with $C_{Max}$ carbons when $n$ carbons of $^{13}C$ are already present due to incorporation from the labeling source ($P_C$ is the "product" correction for carbon). Equation (4) represents the fraction of the $I_{M+n}$ peak intensity that is converted to other isotopologues due to the effects of natural abundance and is expressed as a function of $n$ (the isotopomer number, $S_C$ is the "sum" correction for carbon) [3].

$$S_C(n) = \sum_{k=n+1}^{C_{Max}} P_C(n,k) \tag{4}$$

Equation (5) shows the full correction of the $i$th isotopologue ($I_{M+i}$) by subtracting the natural abundance contributions based on lower mass untainted isotopologue intensities. Here the entire calculation has been divided by the fractional intensity, $1 - S_C(i)$, in order to compensate for natural abundance effects that lower the intensity of the given isotopologue. To calculate the $i$th corrected intensity in the series, the first $i - 1$ intensities must be corrected for natural abundance, this requirement means that correction of a set of isotopologue intensities must be carried out in a sequential fashion starting with $i = 0$ [3].

$$I_{M+i} = \frac{I_{M+i;NA} - \sum_{x=0}^{x<i} I_{M+x} P_C(x,i)}{1 - S_C(i)} \tag{5}$$

The very high mass resolution of the FT-ICR-MS histograms allows $^{15}N$ incorporation to be distinguished from $^{13}C$ incorporation, making the extension of this method to $^{15}N$ labeling trivial, only requiring the replacement of $C_{Max}$ with $N_{Max}^{-}$ (the maximum number of nitrogen atoms in the molecule) and $NA_{13C}$ with $NA_{15N}$. Unfortunately this is not the case when performing corrections on sets of intensities where both $^{13}C$ and $^{15}N$ are incorporated from labeling sources and natural abundance.

### 1.2. Two (or More) Isotope Labels

Using two isotope labeling sources simultaneously creates a two dimensional isotopologue dataset. In general for $n$ labeling sources, the dimensionality of the dataset will be $n$-dimensions. For the case of mixed $^{13}C$ and $^{15}N$ labeling sources, there will be up to $C_{Max} * N^{-}_{Max}$ intensities to correct, each having a unique index reflecting the exact number of $^{13}C$ and $^{15}N$ atoms in the isotopologue. This requires modifications to Equation (5). In particular, the "penultimate" boundary condition of the series must correctly generalize to a multidimensional case. These modifications are represented by Equation (6), which correctly handles the two dimensional penultimate boundary conditions, both in the numerator and denominator. Also, Equation (6) is a correction to a boundary condition error in the previously proposed analytical solution [3].

$$I_{M+i,j} = \frac{I_{M+i,j;NA} - \sum_{x=0;y=0}^{\substack{x\le i;y\le j \\ x\ne i|y\ne j}} I_{M+x,y} P_C(x,i) P_N(y,j)}{\left(1 - S_C(i)\right)\left(1 - S_N(j)\right)} \tag{6}$$

Specifically, to correct the intensity of an isotopologue with $i$ $^{13}C$ atoms and $j$ $^{15}N$ atoms, all intensities indexed within the space from (0,0) to (*i,j*), excluding element (*i,j*) itself, must be considered in the series. We refer to this set as the penultimate set of element ($i,j$). Furthermore, these equations can be extended in a similar way to accommodate any number of labels, although practical considerations of computational efficiency and analytical sensitivity will limit most applications to three isotopes or less. Equation (7) shows the extension of Equation (5) to three labels: $^{13}C$, $^{15}N$, and $^{2}H$.

$$I_{M+i,j,h} = \frac{I_{M+i,j,h;NA} - \sum_{x=0;y=0;z=0}^{\substack{x\le i;y\le j;z\le h\\ x\ne i|y\ne j|z\ne h}} I_{M+x,y,z} P_C(x,i) P_N(y,j) P_H(z,h)}{\left(1 - S_C(i)\right)\left(1 - S_N(j)\right)\left(1 - S_H(h)\right)} \quad (7)$$

## 2. Interleaving Zero Values

In addition to comparing the absolute numerical values of the different methods of calculating the P-correction values, we also wanted to determine if there are any instances where interleaving returns values that the others do not, or vice-versa. The primary driver of the values is the "k − n" term in Equation (3). We therefore examine the values with respect to the value of "k − n". The P correction terms are also plotted as −1 × log10 of the value. Figure S1a highlights the result that for "k − n" values between 0 and 84, both org and choose methods always return comparable values; however, choose returns zero for all k − n > 84, while org returns some non-zero values out to k − n < 115. Choose is being floored to zero due to the value of the exponential k − n on the natural abundance ($0.00015^{85}$); however org returns values, with some of them being zero (Figure S1b). To investigate if a mix of values and zeros should be returned over this range of k − n, the values returned by org were compared with the logReal, and checked if there were any instances of org returning a value different from zero when logReal returns zero (or vice-versa). No instances were found of this happening. To view where values are floored to zero in Python, the values from the log algorithm were plotted, with those values where logReal (the transformation of the log value to real space) returns zero noted, as shown in Figure 4c. The limit of the org method is the same as using logs in the equation and subsequently transforming the real space, at $10^{-323.6}$ (red line in Figure S1c). Past this limit Python floors the values to zero.

**Figure S1.** “k − n” plotted against −1 × log10 of the P values from Equation (3). Zero values are not transformed, and are plotted as zero. (**a**) P-correction values calculated using the “org” method. Green points are those for which both “org” and “comb” return values that are not zero (both work, k − n values of 0 to 84), orange points are those for which “org” returned numerical values but “org” returned zero (choose fails, k − n values of 85 to 115), and purple points are those where both “org” and “comb” returned zero (both fail, k − n > 115). (**b**) P-correction values calculated using “org”. Orange denotes points that may be returning zeros in error (potential problem, 85 < k − n < 115). (**c**) P-correction values generated using the log version of the calculation. Green points are those for which transformation of the value is not zero (return value), purple points return zero (return zero). The red line denotes the limit at which transforming log-values to real space returns zero. It should be noted that the return zero points start at the previously indicated limit of k − n equal to 85.

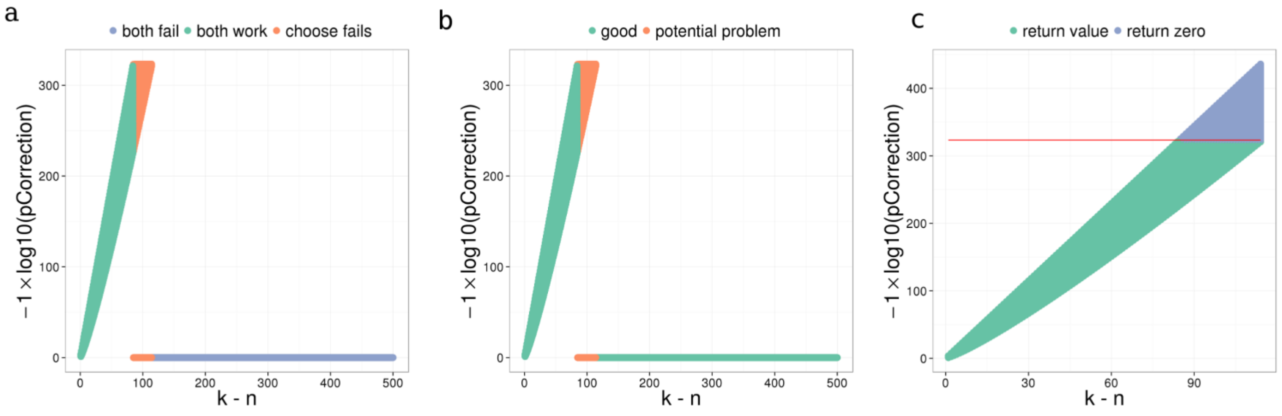


## 3. Python Code to Test P-Correction Methods

Below is the actual Python code that was used to generate the P-correction values using the different methods.

```
from __future__ import print_function
from math import factorial, log10
from scipy.misc import comb

 def NABC_org(a, b, c, na):
 denomax = min(a - b, b)
 numermin = max(a - b, b)
 divisor = 1
 result = 1
 while(a > numermin or divisor <= denomax or b > 0 or c > 0):
   if(a > numermin):
   result *= a
   a -= 1
   if(divisor <= denomax):
```

```
    result /= divisor
    divisor += 1
    if(b > 0):
    result *= na
    b -= 1
    if(c > 0):
    result *= (1 - na)
    c -= 1
  return result

def NABC_log(a, b, c, na):
  denomax = min(a - b, b)
  numermin = max(a - b, b)
  divisor = 1
  result  = 0
  while(a > numermin or divisor <= denomax or b > 0 or c > 0):
    if(a > numermin):
    result += log10(a)
    a -= 1
    if(divisor <= denomax):
    result -= log10(divisor)
    divisor += 1
    if(b > 0):
    result += log10(na)
    b -= 1
    if(c > 0):
    result += log10(1 - na)
    c -= 1
  return result

def NABC_comb(a, b, c, na):
  result = comb(a, b, exact="TRUE") * (na ** b) * ((1 - na) ** c)
  return result

def NABC_comb2(a, b, c, na):
  result = comb(a, b) * (na ** b) * ((1 - na) ** c)
  return result

def choose_set(n, k):
  nk = n-k
  nFact = factorial(n)
  kFact = factorial(k)
  nkFact = factorial(nk)
  return(nFact / (kFact * nkFact))
```

```
def NABC_choose(a, b, c, na):
  result = choose_set(a, b) * (na ** b) * ((1 - na) ** c)
  return result

# <codecell>

fOut = open('./nk_errors.txt', 'w+')
na = 0.00015
imax = 500

whichVals = ["org", "comb", "comb2", "choose", "logReal"]
whichComb = []

for iVal in range(0, len(whichVals)):
    for jVal in range(iVal+1, len(whichVals)):
      whichComb.append(str(whichVals[iVal]) + "_" + str(whichVals[jVal]))

headerStr = ["imax", "n", "k"]
headerStr.extend(whichVals)
headerStr.extend(["log"])
headerStr.extend(whichComb)

headerOut = ','.join(map(str, headerStr))
fOut.write(headerOut)
fOut.write("\n")

for n in xrange(imax):
    for k in xrange(imax, n, -1):
        resultVals = [NABC_org(imax-n, k-n, imax-k, na),
                      NABC_comb(imax-n, k-n, imax-k, na),
                      NABC_comb2(imax-n, k-n, imax-k, na),
                      NABC_choose(imax-n, k-n, imax-k, na)]
        logVal = NABC_log(imax-n, k-n, imax-k, na)
        logReal = 10**logVal

        resultVals.append(logReal)

        diffVals = []

        for iDiff in xrange(0, len(resultVals)):
          for jDiff in xrange(iDiff+1, len(resultVals)):
            diffVals.append(resultVals[iDiff] - resultVals[jDiff])

        outVals = [imax, n, k]
        outVals.extend(resultVals)
        outVals.extend([logVal])
        outVals.extend(diffVals)

        outStr = ','.join(map(repr, outVals))
```

```
        fOut.write(outStr)
        fOut.write("\n")
    #print(outStr)
fOut.close()
```

**References**


1. Van Winden, W.; Wittmann, C.; Heinzle, E.; Heijnen, J. Correcting mass isotopomer distributions for naturally occurring isotopes. *Biotechnol. Bioeng.* **2002**, *80*, 477–479.
2. Zhang, X.; Hines, W.; Adamec, J.; Asara, J.; Naylor, S.; Regnier, F. An automated method for the analysis of stable isotope labeling data in proteomics. *J. Am. Soc. Mass Spectrom.* **2005**, *16*, 1181–1191.
3. Moseley, H.N. Correcting for the effects of natural abundance in stable isotope resolved metabolomics experiments involving ultra-high resolution mass spectrometry. *BMC Bioinformatics* **2010**, *11*, doi:10.1186/1471-2105-11-139.
